# Bcl-2 over-expression fails to prevent age-related loss of calretinin positive neurons in the mouse dentate gyrus

**DOI:** 10.1186/1750-1326-1-9

**Published:** 2006-08-22

**Authors:** Mingbo Han, Frank Schottler, Debin Lei, Elizabeth Y Dong, Alexander Bryan, Jianxin Bao

**Affiliations:** 1 Department of Otolaryngology, Washington University in St. Louis School of Medicine, St. Louis, MO, USA; 2 Center for Aging, Washington University in St. Louis School of Medicine, St. Louis, MO, USA

## Abstract

**Background:**

Cognitive performance declines with increasing age. Possible cellular mechanisms underlying this age-related functional decline remain incompletely understood. Early studies attributed this functional decline to age-related neuronal loss. Subsequent studies using unbiased stereological techniques found little or no neuronal loss during aging. However, studies using specific cellular markers found age-related loss of specific neuronal types. To test whether there is age-related loss of specific neuronal populations in the hippocampus, and subsequently, whether over-expression of the B-cell lymphoma protein-2 (Bcl-2) in these neurons could delay possible age-related neuronal loss, we examined calretinin (CR) positive neurons in the mouse dentate gyrus during aging.

**Result:**

In normal mice, there was an age-related loss of CR positive cells in the dentate gyrus. At the same region, there was no significant decrease of total numbers of neurons, which suggested that age-related loss of CR positive cells was due to the decrease of CR expression in these cells instead of cell death. In the transgenic mouse line over-expressing Bcl-2 in neurons, there was an age-related loss of CR positive cells. Interestingly, there was also an age-related neuronal loss in this transgenic mouse line.

**Conclusion:**

These data suggest an age-related loss of CR positive neurons but not total neuronal loss in normal mice and this age-related neuronal change is not prevented by Bcl-2 over-expression.

## Background

Decline of cognitive functions, such as learning and memory, is often associated with aging, which plays a crucial determinant of the quality of life in elderly population [1-4]. Normal age-related deficits in learning and memory resemble those caused by damage to the hippocampus. In the hippocampus, electrophysiological studies demonstrated age-related deficits in the induction and maintenance of long-term potentiation (LTP), and lower thresholds for potentiation and long-term depression [5-7]. Despite such functional evidence for age-related dysfunction in the hippocampus, the cellular and molecular bases of this decline are still unclear. Autopsy and magnetic resonance imaging-based volumetric measurements in normal elderly humans have shown hippocampal shrinkage [8,9]. This atrophy could be theoretically accounted for by age-related neuronal loss in the hippocampus. Early studies in many species did report neuronal loss of hippocampal principal cells during aging [for review, see [11]]. Subsequently, based on unbiased stereological techniques to estimate neuron number, the overall results suggested that there was no widespread hippocampal cell loss in both human and rodent models during aging [12-18]. Despite maintenance of total neuron number, there may be a loss of subpopulation of neurons. The design-based stereological analysis may be unable to detect changes in specific neuronal populations because most of these studies focused on the total neuronal population. Indeed, studies using specific molecular markers clearly showed age-related decrease of specific neuronal populations, which could contribute to age-related functional decline of brain functions [19-22]. However, one detailed study found that age-related loss of hippocampal interneurons positive for glutamate decarboxylase-67 (GAD-67, a key synthesizing enzyme for GABA) was due to loss of GAD-67 expression rather than neuronal loss [23]. Thus, it remained unclear whether there was any age-related neuronal loss.

If there was age-related neuronal loss or age-related changes of specific neuronal types, the next important question is whether these age-related neuronal changes could be prevented. One particular protein, the proto-oncogene B-cell lymphoma protein-2 (Bcl-2), has been shown to prevent both apoptotic and necrotic neuronal death [24]. Bcl-2 gene is originally identified in B-cell lymphoma where its deregulated expression protects cells from apoptosis [25-27]. In the nervous system, Bcl-2 is detected and had been shown to protect neurons from undergoing apoptosis during early development or neuronal insults by a wide variety of stimuli, including growth factor deprivation and oxidation stress [28-31]. However, Bcl-2 over-expression does not prevent mutant neurofilament-mediated motor neuron degeneration [32]. In the cerebellum, over-expression of the human Bcl-2 transgene with a neuron-specific enolase (NSE) promoter initially increase the number of Purkinje cells by preventing neuronal death during development, but subsequently cannot prevent age-related loss of these neurons [33]. In the hippocampus, Bcl-2 expression is decreased during aging [34]. It is still unknown whether Bcl-2 over-expression can prevent age-related loss of specific neuronal populations in the hippocampus.

In the dentate gyrus of mouse hippocampus, several high-affinity cytosolic calcium binding proteins such as parvalbumin, calbindin, and calretinin (CR) have been shown to be excellent chemical markers for certain interneurons (35–41). A majority of interneurons in the hilus are positive for CR [35-38]. Based on detailed histological examinations, these CR positive neurons in the hilus are mossy neurons [39-41]. CR is also an early and specific marker of newly generated adult-born neurons located near the edge of the hilus at the subgranule zone (SGZ). Its expression can be detected as early as one day after the neuron is born and last less than six weeks [37]. Our preliminary studies found over-expression of Bcl-2 transgene in this neuronal population from mice expressing the human Bcl-2 gene under the control of the neuron-specific enolase promoter (the NSE73a line). Based on these previous detailed studies, we sought to focus on this population of CR positive interneurons to test whether there was age-related loss of interneurons in the mouse dentate gyrus, and whether Bcl-2 over-expression could prevent age-related loss of this neuronal population.

## Results

### Expression of human Bcl-2 in mouse hippocampus

**Figure 1:**
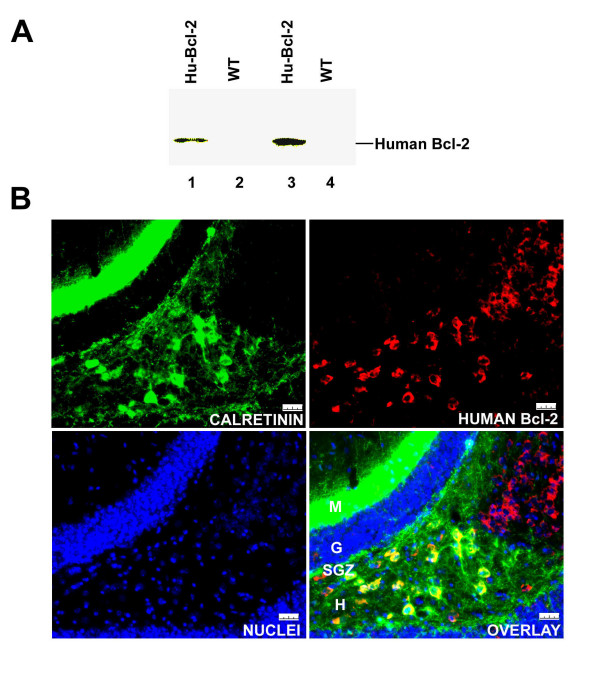
**Over-expression of Human Bcl-2 in the mouse hippocampus**. A) Western-blots of the hippocampal lysates from 18 month-old C57BL/6J (WT, two mice) and NSE73a transgenic mice (Hu-Bcl-2, two mice). B) Co-localization of the transgenic Bcl-2 and CR in the hippocampus. Four layers of the mouse dentate gyrus are labeled on the overlay: M, molecular layer; G, granule cell layer; SGZ, sub-granule cell zone; and H, Hilus. The whole calibration length is 25 μm.

To examine the expression of human Bcl-2 gene in the hippocampus of the transgenic mice, we prepared lysates from hippocampuses of 18 month-old transgenic and wild type mice. These samples were analyzed by Western blotting using a monoclonal antibody that does not cross-react with mouse Bcl-2. Human Bcl-2 was detected in the hippocampal lysates from transgenic mice (Fig. 1A). Although immunohistochemistry from previous studies indicated that expression of the human Bcl-2 was restricted to neurons in this transgenic mouse line due to the use of neuron-specific enolase promoter [33,42], the expression pattern of human Bcl-2 in the dentate gyrus was unknown. We found that the transgenic human Bcl-2 was expressed mostly in the hilus and co-localized with CR positive interneurons (Fig. 1B). The expression of human Bcl-2 was also found in CR positive cells at the SGZ. In the granular cell layer, the transgene expression is below the level to be detected (Fig. 1B).

### Age-related decrease of CR positive neurons in the dentate gyrus

**Figure 2:**
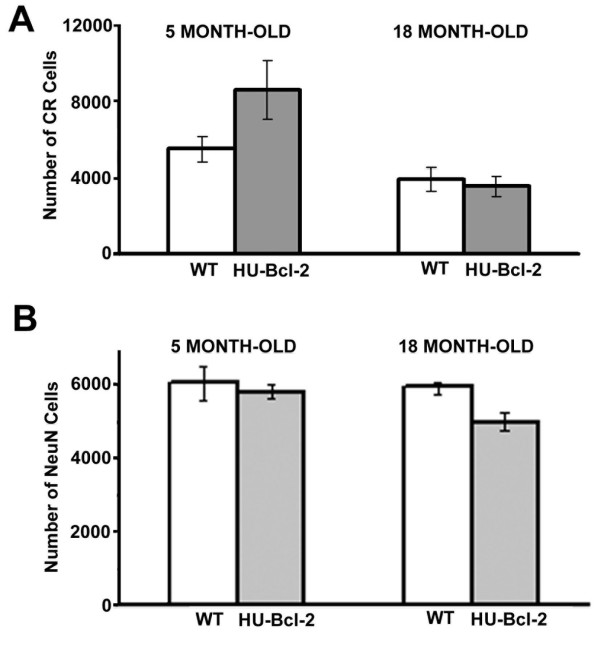
**Analysis of age-related neuronal loss in the mouse dentate gyrus**. Mean number of CR positive neurons or total neurons were estimated in both wild-type (5 month-old, n = 4; 18 month-old, n = 5) and NSE73a transgenic mice (5 month-old, n = 3; 18 month-old, n = 3). A) Mean number of CR positive neurons (+/- SD) estimated in the SGZ and hilus of the mouse dentate gyrus. Based on two-way ANOVA, There was a statistical difference between two age groups (P = 0.003), but there was no difference between two genotypes (P = 0.144), and there was not a statistically significant interaction between genotype and age (P = 0.07). B) Mean number of NeuN positive neurons (+/- SD) estimated in the same region as in (A). Based on two-way ANOVA, There was not a statistical difference between two age groups (P = 0.142), not a difference between two genotypes (P = 0.076), and not a significant interaction between genotype and age (P = 0.266).

Since almost all co-localization of CR and Bcl-2 over-expression were found in the hilus and the SGZ, the number of CR positive neurons in these areas was estimated for both control (normal C57BL/6J mice) and Bcl-2 transgenic mice under C57BL/6J genetic background with an unbiased stereology approach. At five-month old, in comparison with the normal C57BL/6J mice, the number of CR positive neurons within the hilus and SGZ exhibited over 56% increase in the transgenic mice, which was consistent with previous reports that more neurons were found in this transgenic mouse line with Bcl-2 over-expression [33,42,43]. However, at 18 month-old, there were no differences in the number of CR positive neurons between the control and transgenic mice. In comparison with mice at five-month old, there was a significant decrease of CR positive neurons for both control and transgenic mice at 18 months old (P = 0.003, F = 14.752, DF = 1, two-way ANOVA). For the control mice, there was about 29% reduction of CR positive neurons in the dentate gyrus from 5 to 18 month-old; and for the transgenic mice, there was about 59% reduction (Fig. 2A). Based on the two-way ANOVA testing, there was no difference between two genotypes (P = 0.144, F = 2.474, DF = 1), and also no significant interaction between genotype and age (P = 0.07, F = 4.039, DF = 1).

Because one detailed study clearly demonstrated that age-related reduction in the number of GAD-67 positive interneurons were due to age-related loss of GAD-67 expression rather that age-related neurons loss in the hippocampus [23], we estimated the total neuronal numbers in the same brain sections using the unbiased stereology after the immunostaining of a neuronal marker, NeuN. There was no age-related change in total neuronal numbers for normal C57BL/6J mice during aging (P = 0.43, one-way ANOVA). Surprisingly, there was a significant reduction (14%) of total neurons for the transgenic mice during aging (P = 0.04, one-way ANOVA). Based on the two-way ANOVA testing, there is no difference between control and transgenic mice (P = 0.076, F = 4.14, DF = 1), and there was no significant interaction between genotype and age (P = 0.266, F = 1.431, DF = 1). These results suggested that, in the control mice, age-related loss of CR positive neurons was due to a decrease of CR expression; while in the transgenic mice; age-related loss of CR positive neurons was due to both an age-related decrease of CR expression and age-related neuronal loss.

### Proliferation rate of adult neurogenesis in the dentate gyrus during aging

**Figure 3:**
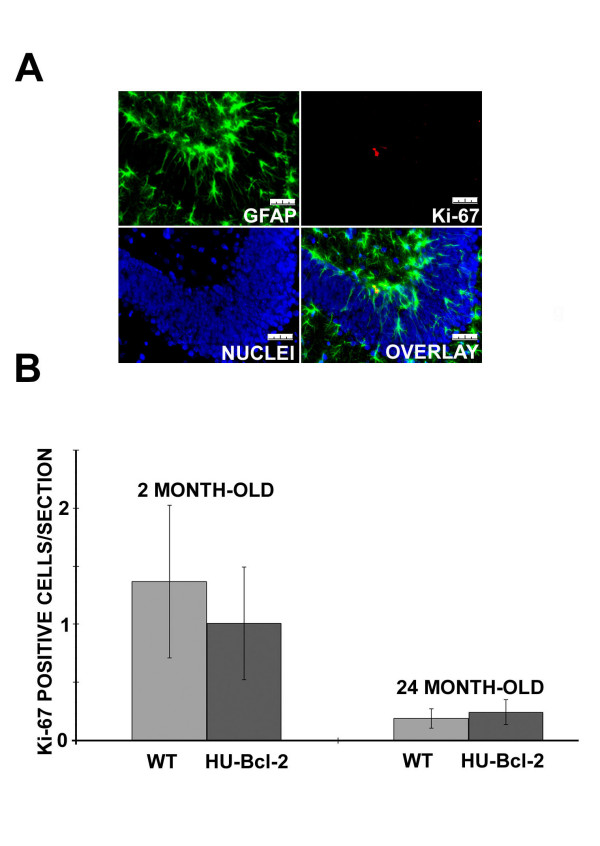
**Proliferation rate of adult neurogenesis using Ki-67 as a marker**. A) One example of GFP (green) and Ki-67 (red) in a 2 month-old mouse dentate gyrus. The whole calibration bar is 25 μm. B)Semi-quantification of Ki-67 positive cells in both wild-type (2 month-old, n = 3; 24-month-old, n = 3) and NSE73a transgenic mice (2 month-old, n = 3; 24-month-old, n = 3) (+/- SE). Based on two-way ANOVA, There was a statistical difference between two age groups (P = 0.004), but there was no difference between two genotypes (P = 0.541), and also no a statistically significant interaction between genotype and age (P = 0.410).

However, there were several possible factors that could influence the above findings. Since there is a consistent adult neurogenesis in the SGZ, and one of these possibilities was that Bcl-2 over-expression might change the proliferation rate of adult neurogenesis during aging. For example, an accelerated decrease of the proliferation rate would mask a delay of age-related decrease of CR expression due to Bcl-2 over-expression. We examined the proliferation rate of adult neurogenesis in both wild type and transgenic mice with Ki-67 as a proliferate marker. Although there was a dramatic decrease of the number of Ki-67 positive cells from 2 month-old to 24 month-old mice (P = 0.004, F = 16.604, DF = 1, two-way ANOVA), no statistical difference was found between wild type and transgenic mice (Fig. 3, P = 0.541, F = 0.407, DF = 1, two-way ANOVA). There was also no significant interaction between genotype and age (P = 0.410, F = 0.754, DF = 1, two-way ANOVA). Thus, over-expression of human Bcl-2 did not affect the rate of adult neurogenesis during aging.

### Molecular characterization of CR positive neurons in the dentate gyrus over-expressing Bcl-2

**Figure 4:**
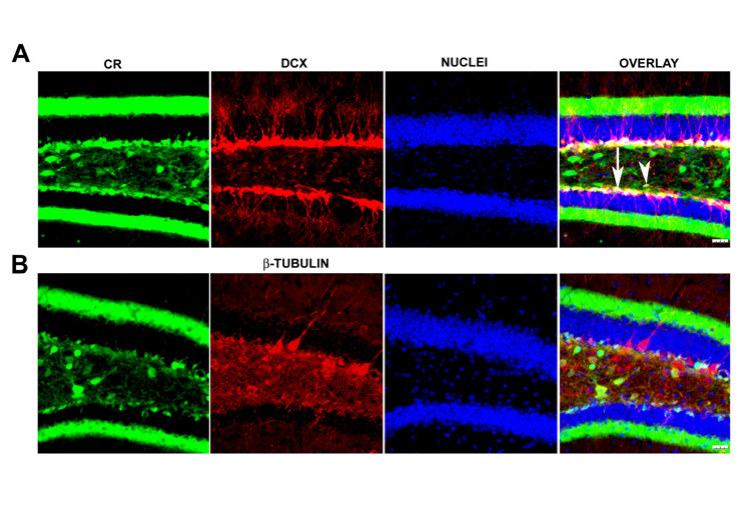
**Characterization of CR positive cells in the dentate gyrus**. A) Co-localization of CR with DCX at the SGZ. No co-localization was observed in the hilus. At the overlay panel, the long arrow points an example of late progenitor cells with processes and the short arrow an early progenitor cell without any apical processes. B) Co-localization of CR with β-tubulin in the dentate gyrus. β-tubulin is a neuronal specific molecular marker for mature neurons. Almost all CR positive cells were co-localized with β-tubulin in the hilus. The whole calibration bar is 25 μm.

Another possibility that could complicate our conclusions was that Bcl-2 over-expression disrupted CR expression patterns in the dentate gyrus. In the normal mouse dentate gyrus, there are two populations of CR-positive neurons in this region, CR positive neurons in the hilus are mature hilar mossy neurons [40,41], while CR positive cells at the SGL are early postmitotic neurons characterized by the transient CR expression, which starts one day after cell division and ends around six weeks after [37]. Doublecortin (DCX) is a marker for progenitor and early newborn neurons [44]. Thus, it should not be detected in CR positive neurons in the hilus but should be present in CR positive neurons in the SGZ. A similar pattern was preserved in the transgenic mice (Fig. 4A). Furthermore, at the SGZ, there are two types of DCX and CR double-positive progenitor cells: early progenitor neurons without any apical processes (less than 7 days old, the short arrow in the Overlay) and late progenitor neurons with processes projected into the granule cell layer (7 days and older, the long arrow in the Overlay) [44]. These two types of progenitor cells were also found in the transgenic mice (Fig. 4A). Using β-tubulin, a mature neuronal maker, we found CR and β-tubulin double-positive neurons in the hilus, consistent with CR positive cells in the hilus as mature mossy neurons (Fig. 4B). Only very few CR positive cells were co-localized with β-tubulin at the SGZ. These expression patterns were the same as reported in the normal mice [39-41]. We did not find any difference in these co-localization patterns between transgenic and normal mice. Thus, Bcl-2 over-expression did not lead to obvious abnormal CR expression patterns in the dentate gyrus.

### Distribution of CR positive neurons in dentate gyrus during aging

**Figure 5:**
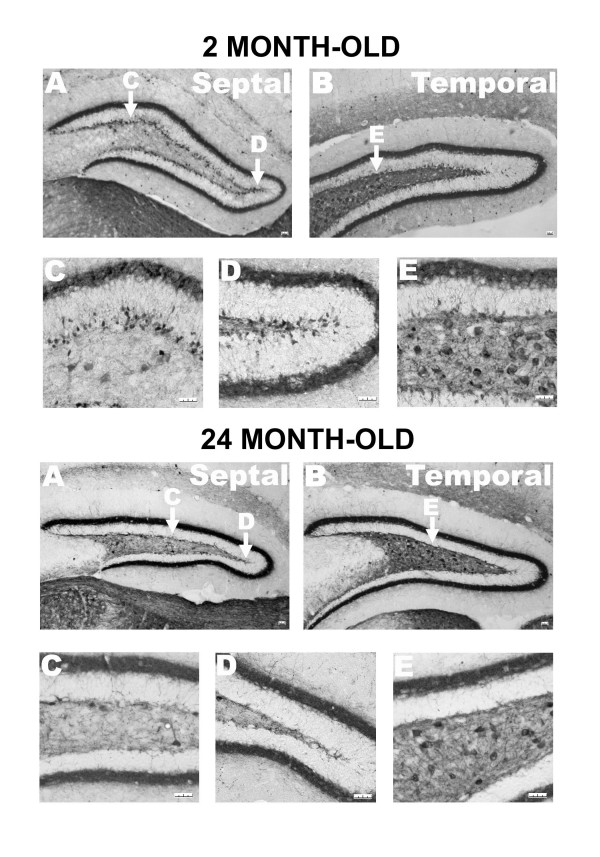
**Spatial distribution of CR positive cells in the dentate gyrus of NSE73a transgenic mice**. In the dentate gyrus of 2 month-old mice, the number of CR positive cells distributed at the SGZ was much higher in the septal region (A) that in the temporal region (B) of the dentate gyrus, and the number of the hilular CR positive cells was much higher in the temporal region than in the septal region. The same distribution pattern held in the dentate gyrus of 24 month-old mice although the number of CR positive cells at both hilus and SGZ was less that the number in 2 month-old mice. The whole calibration bar is 25 μm.

Since the dentate gyrus is a 3-D structure, it was possible that Bcl-2 over-expression could disrupt the distribution of CR neurons in this 3-D structure. Although it should not affect the final conclusions because we used the unbiased stereological counting method, it could introduce large errors and reduce the sensitivity of our approach. We thus examined age-related changes of CR neurons in the dentate gyrus from septal (dorsal) to temporal (ventral) regions in the transgenic mice. The distribution of CR positive neurons was very similar if not the same as the normal mice. At the septal region, one obvious difference between young (2 month-old) and old (24 months-old) mice was a dramatic decrease of CR positive neurons at the SGZ in the dentate gyrus (Fig. 5). At the temporal region, similar to previous findings [35], more CR positive neurons were found in the hilus, compared to the septal region (Fig. 5).

### Changes of CR positive neurons at the SGZ during aging

**Figure 6:**
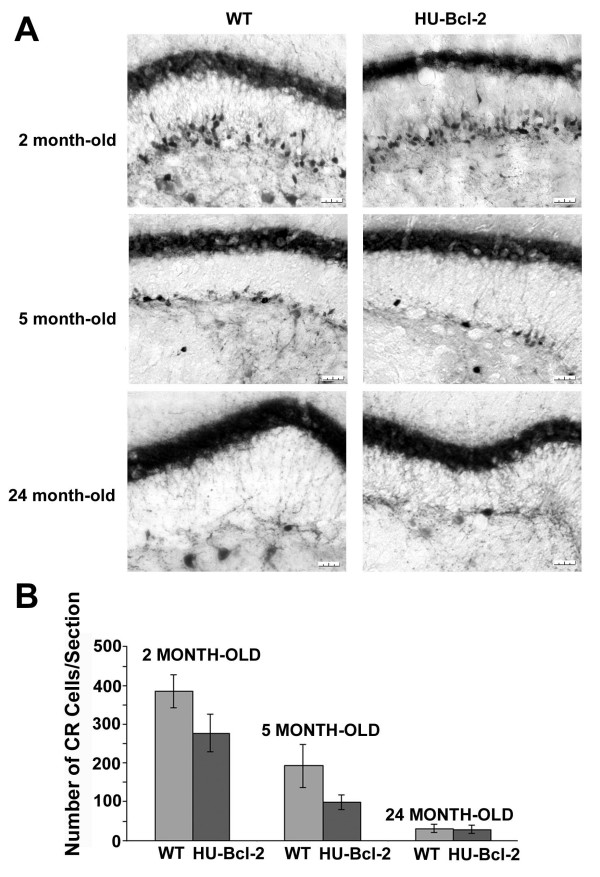
**Age-related decease of CR positive cells at the SGZ**. A)Dramatic decrease of CR positive cells at the SGZ during aging. The whole calibration bar is 25 μm. B) Semi-quantification of CR positive cells at the SGZ in both wild-type (total 9 mice, 3 for each age group) and NSE73a transgenic mice (total 9 mice, 3 for each age group) (+/- SE). Based on two-way ANOVA, There was a statistical difference between two age groups (P = 0.001), between two genotypes (P = 0.002), and a significant interaction between genotype and age (P = 0.049).

Based on the above findings, we focused on the CR positive neurons at the SGZ of the septal area among three age groups (2, 5, and 24 month-old). In both wild type and transgenic mice over-expressing human Bcl-2, there was a dramatic decrease of CR positive neurons at the SGZ, and this could be observed as early as in the 5 month-old mice (Fig. 6A). Semi-quantitatively, there was a dramatic decrease of CR positive neurons at the SGZ during aging (P = 0.001, F = 102.341, DF = 2, two-way ANOVA), and there was a statistical difference between wild type and transgenic mice (P = 0.002, F = 15.379, DF = 1, two-way ANOVA). Compared to the wild type mice, there were fewer CR positive neurons at the SGZ from 2 and 5 month-old transgenic mice (Fig. 6B). Thus, an increase of CR positive neurons in the dentate gyrus of the Bcl-2 transgenic mice at five months of age (Fig. 2A) could be only due to an increase of mossy neurons in the hilus, and over-expressing of human Bcl-2 gene could not delay age-related decrease of CR expression in mossy neurons at later stages (over 18 month-old).

## Discussion

The results presented here reveal a consistent age-related loss of CR positive neurons in the mouse dentate gyrus. However, in normal mice, this age-related loss is due to a decrease of CR expression without significant neuronal loss. Over-expressing Bcl-2 does not prevent this age-related loss of CR positive neurons. Unexpectedly, Bcl-2 over-expression leads to age-related neuronal loss in the dentate gyrus.

CR positive neurons in the mouse dentate gyrus are well characterized [37,39-41]. At the SGZ, CR is transiently expressed in newly generated postmitotic neurons. Thus, CR is a useful marker to measure adult neurogenesis [45]. Consistent with the previous studies showing an age-related decrease of neurogenesis in the dentate gyrus [46], we found a clear age-related loss of CR-positive cells at the SGZ. Interestingly, over-expression of Bcl-2 decreases this population of CR positive cells around two to five month-old. This finding seems to contradict the previous finding made using DCX as a molecular maker in the same transgenic mice [47], which found a significant increase of DCX positive cells in the region including the granule cell layer and SGZ around two-month old. There are two obvious differences between our study and their study. First of all, different molecular markers were used (DCX vs. CR), and DCX is expressed in both proliferating progenitor cells and newly generated postmitotic neurons. Second, different areas were investigated: both SGZ and granular cell layer in their study and the SGZ only in our study. However, we found a high level of co-localization for these two markers in the SGZ and only a few cells positive for both markers at the granular cell layer. Therefore, there may be other factors contributing to the difference, for example, we found that it was hard to precisely count DCX positive cells because not only cell bodies but also extensive processes were labeled. With CR as a molecular marker, we were able not only to easily count the positive cells but also to observe a similar trend between control and transgenic mice at three different age groups (2, 5 and 24 month-old). Based on these data, and other control experiments we did, we have concluded that Bcl-2 over-expression could not delay age-related decrease of adult neurogenesis.

In the hilus, CR positive neurons located at the ventral part of the hilus are mossy cells, which receive innervations from mossy fibers of granule cells in the dentate gyrus [48]. Mossy neurons in turn project to the ipsi- and contra-lateral inner molecular layer and make excitatory synapses on proximal dendrites of granule cells [49]. Adult neurogenesis does not contribute to this population of neurons [37]. With the unbiased stereological approach to estimate total CR positive cells in the region including both the SGZ and hilus, we found more CR positive neurons at 5 month-old transgenic mice compared to the control mice. Because the number of CR positive cells was less at the SGZ in the transgenic mice, it is reasonable to assume that there were more CR positive cells (or mossy cells) in the hilus of the transgenic mice. Because the total number of neurons in the hilus was similar between the transgenic and control mice, Bcl-2 over-expression may either selectively increase CR positive neurons or up-regulate CR expressions in the hilus, which was hard to distinguish in the present study. Another caveat was that the immunoreactive intensity for NeuN was not as strong as CR; therefore, there was a tendency to underestimate the total neuronal number by using NeuN as a marker. However, using the same transgenic line, one previous study found an early increase of the Purkinje cells and a return to near the normal level during aging [33]. Similar to their findings in the cerebellum, we found no difference in the number of CR positive cells between the control and transgenic mice at 18-month old, which suggests that Bcl-2 over-expression can not prevent age-related loss of CR positive cells. Furthermore, Bcl-2 over-expression leads to age-related neuronal loss while there is no significant neuronal loss in the control animals during aging. Since this transgenic line shows a deficit of allocentric navigation in the water maze and a decrease of LTP amplitude [50], one possible reason for this observation is that Bcl-2 over-expression at early development stages could result in abnormal neuronal connections, which leads to abnormal neuronal activities, and subsequently accelerated neuronal loss during aging.

In conclusion, we present strong evidence for age-related loss of CR positive cells in the mouse dentate gyrus and this loss is not prevented by Bcl-2 over-expression. The age-related decrease of CR is likely to contribute to age-related functional decline of nervous system. The absence or reduction of CR in mossy neurons has been associated with a complete blockade of dentate LTP induction in mice [51,52]. The cause of this blockade may due to decreased calcium buffering capacity in hillar mossy cells, which leads to abnormal changes in synaptic transmission. A similar change in the mouse dentate gyrus may happen during aging, which could be one of the cellular mechanisms underlying age-related decrease of the dentate LTP. Furthermore, age-related decrease of CR expression could also deprive these neurons of the capacity to buffer intracellular calcium and thus leave them vulnerable to calcium excitoxicity during aging.

## Materials and methods

### Animals

C57BL/6J mice and mice expressing the human Bcl-2 gene under the control of the neuron-specific enolase promoter (the NSE73a line) were bred in the CID animal care facility at Washington University in St. Louis. All experimental protocols were approved by the Institutional Animal Care and Use Committee (Washington University/CID). In order to reduce the possibility of effects of other genes co-segregating with the bcl-2 transgene, the Bcl-2 over-expressing transgenic mice were backcrossed to C57BL/6J for at least 8 generations prior to the onset of our studies. The genotype of each mouse was determined by tail-clip DNA analysis using PCR. A total of 24 C57BL/6J and 21 transgenic mice were used for the data analysis. The detail usage of animals for each experiment group was listed (Table 1).

**Table 1:** Animal numbers in each experiment group.

Groups	Age (months)	Number of C57BL/6J mice	Number of NSE73a mice	Subtotal
Stereological estimation of CR and NeuN positive neurons	5	4	3	7
	18	5	3	6
Semi-quantitation of CR neurons	2	3	3	6
	5	3	3	6
	24	3	3	6
Semi-quantitation of Ki67 cells	2	3	3	6
	24	3	3	6

Total		24	21	45

### Tissue preparation and immunohistochemistry

Mice were transcardially perfused with 2% paraformaldehyde and 2% glutaraldehyde in 0.1 M sodium phosphate, PH 7.6. The brains were removed and kept in the fixative overnight and then transferred into 30% sucrose. Tissue samples were immersed in OCT compound (Sakura Finetek USA, Torrance, CA) and frozen on dry ice. Cryostat sections were cut. The sections were stored at – 20 C in cryoprotectant with 25% ethylene glycol, 25% glycerin, and 0.05 M phosphate buffer. Free-floating immunohistochemistry was used for all sections. Two different immunostaining methods were applied: the peroxidase method (ABC system, Vectastain, Vector Laboratories) for anti-CR immunostaining (rabbit anti-CR, 1:1000, Chemicon) on 20 μm sections; and immunoflurescent labeling for CR, human Bcl-2 (1:1000, PharMingen, San Diego, CA), neuron-specific nuclear antigen (NeuN) (1:500, Chemicon), doublecortinin (1:1000, Santa Cruz Lab.), Ki67 (1:500, BD Biosciences Pharmingen), and β-tubulin (1:500, Covance) on 40 μm sections. Fluorescent sections were mounted with DAP (to label the nuclei) in polyvinyl alcohol with diazbicyclo-octans as antifading agent.

### Stereology

To precisely examine possible age-related neuronal loss in the hilus during aging, we estimated the number of CR or NeuN positive neurons in the hilus by using the optical volume fractionator procedure. This method employs a design-based, systematically random, multilevel sampling protocol that combines the optical dissector and fractionator methods to estimate subregion volume and cell number. The area and distribution of the dissector counting frames employed were dependent upon the region and cell type under examination. For counting CR or NeuN positive cells, the sampling grid area was 120 × 120 μm, the dissector size for each area was 50 × 50 μm using the 100 × oil immersion objective lens, and the dissector height was 4 μm. The volume was estimated by the product of the summed volume of a systematic sub-sample (every 4^th ^section) throughout the entire structure. The average section thickness was calculated from the section thickness at each position over the sections where a dissector was systematically placed. Since counting efficiency (the number of cells to be counted in each subsection) is dependent on the counting error and biological variability, a random mix of five young and old animals was used to determine the counting efficiency (CE) by calculating the observed coefficient of error and the observed coefficient of variation on relatively thick (40 μm) sections. The average CE for this study was 0.012. The most difficult obstacle with this approach was outlining the hilus based on the polymorphism nuclear regions between the blades of the hilus and the granular cell layer. We failed to consistently outline the hilus (theoretically two nuclei layers away from the granular cell layer) after numerous preliminary experiments. However, we were able to consistently outline the hilus and SGZ together based on the staining pattern of nuclei and CR immunostaining. Thus, we first estimated the total cell numbers of CR and NeuN positive neurons in the region including both the hilus and SGZ with the optical volume fractionator procedure. We then semi-quantitatively estimated the number of CR positive neurons in the SGZ since the optical fractionator cannot be used at the SGZ due to its thickness (only one- to two-cell thick). For this semi-quantification, images of all brain sections from the whole brain were taken under a dissection scope after CR immunostaining. Ten sections with similar dorsoventral level from each were selected among different age groups. For a given section, all CR positive neurons in the SGZ at the dorsal dentate gyrus were counted by an observer blind to the experimental conditions. Cell counts were then summed across the ten sections and divided by ten. Similar approaches were used for counting of Ki-67 positive cells in the SGZ.

### Statistical analysis

The procedures based on West [53] were employed for estimating the observed coefficient of variation and observed coefficient of error in the estimations. Two-way ANONA analysis of variance (SigmaPlot software) was employed to evaluate differences among different age, genotype, and the interaction between age and genotype. Data are presented as mean +/- standard deviation (SD). A p-value less than 0.05 were considered statistically significant.

## Abbreviations

Bcl-2, B-cell lymphoma protein-2; CR, calretinin; DCX, doublecortin; GAD-67, glutamate decarboxylase-67; LTP, long-term potentiation; NSE, neuron-specific enolase; SD, standard deviation; SGZ, subgranular zone;

## Authors' contributions

MH, FS, and DL carried out the experiments, and contributed to the design and analysis of the data. EYD and AB carried out the experiments. JB contributed to the conception, design, and analysis and interpretation of the data, and was responsible for manuscript preparation. All authors read and approved the final manuscript.
